# Factors affecting job satisfaction and commitment among medical interns in Malawi: a cross-sectional study

**DOI:** 10.11604/pamj.2015.21.174.6511

**Published:** 2015-07-03

**Authors:** Andrew Alinafe Mataya, Marcia Eugenia Macuvele, Takudzwanashe Gwitima, Adamson Sinjani Muula

**Affiliations:** 1 Department of Community Health, College of Medicine, University of Malawi, P/B 360 Chichiri, Blantyre 3, Malawi

**Keywords:** Medical internship, Malawi, job satisfaction, job commitment

## Abstract

**Introduction:**

the University of Malawi's College of Medicine produces almost all of the medical interns working at Malawi's two largest public hospitals: Queen Elizabeth Central Hospital in Blantyre, and Kamuzu Central Hospital in Lilongwe. To earn full medical council registration, new graduates must complete an 18-month internship at either site. This study attempted to determine general levels of job satisfaction and commitment among Malawian medical interns, and to rank categorical factors according to impact on intern job satisfaction and commitment. These factors were also examined in terms of influence on bringing about an intern strike in late 2010, early 2011.

**Methods:**

sixty-one of 70 interns working during the study period completed a job satisfaction survey. Questionnaire items addressed ten “factors”: (1) hours and pay, (2) work content, (3) working conditions, (4) relationships with senior staff, (5) workplace relationships with peers, (6) educational environment, (7) internal factors, (8) quality of life, (9) autonomy and self-worth, and (10) future career prospects. Analysis for this cross-sectional study included quantification and comparisons of overall and category-specific satisfaction levels, using ANOVA and Student's t-test.

**Results:**

sixty-nine percent then 57% of interns identified themselves as satisfied at the beginning and end of the questionnaire, respectively; 97% of the interns indicated job commitment at both instances of asking. Factors influencing job satisfaction most strongly were those that participants were least satisfied with.

**Conclusion:**

future research and interventions aimed at improving intern job satisfaction in Malawi should focus on assessing and improving remuneration, working hours, and physical working conditions.

## Introduction

The period immediately after graduation from medical school is supposed to be the time when young doctors consolidate a positive attitude towards clinical work, their relationships with colleagues, future career directions, and job satisfaction [1]. The internship period, however, has been identified as perhaps the most stressful part of a medical career [1]. A number of issues serve to undermine the formative nature of the medical internship, including long work hours; poor working conditions; expectations about work content, compensation, and educational opportunities; and support (or lack thereof) from senior medical staff, nurses, other allied health professionals, and support staff [2]. As a result of these and other factors, it has been found that doctors' overall satisfaction levels are at their lowest in the first year after graduation from medical school [3], and that up to 19% of medical interns have some degree of psychological morbidity [4]. Most of these findings come from the abundance of literature related to junior doctor job satisfaction in the United States or Europe [2, 4-7], for example. The corresponding picture in Africa, where the prevalence of psychological morbidity among medical interns may be even higher [1], remains much less clear.

### Study context

The University of Malawi's College of Medicine (COM), in Blantyre, is Malawi's only medical school, and it produces almost all of the medical interns working at Malawi's two largest public hospitals: Queen Elizabeth Central Hospital (QECH) in Blantyre, and Kamuzu Central Hospital (KCH) in Lilongwe. These are the two main teaching hospitals in the country and Malawian doctors must complete an 18-month internship at either site to obtain full registration with the Medical Council of Malawi. The importance of intern job satisfaction came to a head in Malawi when interns at QECH took part in a strike in late 2010 into early 2011. This study was designed to determine general levels of job satisfaction and commitment among Malawian medical interns, and to rank categorical factors according to how strongly they influence intern job satisfaction and commitment in Malawi. These factors were also examined in terms of their roles in bringing about the aforementioned intern strike.

### Rationale

Job satisfaction is undoubtedly an important determinant of physician retention. In contrast to the lack of publications related to job satisfaction among African physicians, there is an already significant and growing body of literature discussing the related topic of migration of physicians out of Africa [8-12]. It has been suggested that physician “brain drain” is currently a minor problem in Malawi [8], but Malawi still only has a total physician population of about 700 serving a general population of about 15 million [9, 13]. Yes, the establishment of the COM has improved physician numbers in Malawi, but any efforts to maximise retention can be deemed justifiable with the current doctor-to-patient ratio statistics, especially in the view of gradually phasing out dependency on expatriate physicians [10, 11]. Maximising retention is also of financial importance, as highlighted by the finding that the amount of lost investment returns for a single doctor who migrates out of Malawi and serves for 30 years ranges from about USD $433,493 to USD $46 million [12]. Notably, these losses can be considered underestimates because they did not take into account the costs of running the COM [10].

An important implication of this study not to be overlooked is the link between physician job satisfaction and commitment and physician happiness and mental health. Physician unhappiness can lead to disruptive behaviour, burnout, health problems, addiction, depression, and failed relationships [14], all of which can lead to poor patient outcomes and have a negative impact on public health. This negative impact can occur insidiously over the long term, through poor performance, medical errors, and attrition, or overtly and abruptly, through industrial action, as was recently experienced at QECH.

## Methods

After approval was granted by the College of Medicine Research and Ethics Committee (COMREC) in August of 2011, data were collected for this cross-sectional study from 13 to 23 December 2011 at QECH in Blantyre and KCH in Lilongwe. Verbal permission from the heads of the respective departments at the two hospitals was obtained before data were collected using a six-part written questionnaire, which was to be self-administered by each intern and returned to the study team within the ten-day data collection period.

An attempt was made to recruit all interns from the 2010 and 2011 COM Bachelor of Medicine, Bachelor of Surgery (MBBS) graduation cohorts into the study. Subjects were identified from their respective COM graduation lists and intern rotation schedules, which were accessible from the COM central registry and faculty dean's offices, respectively. It was determined, from the available documentation, that there were 70 interns working at the two hospitals during the data collection period, and a questionnaire was distributed to each intern along with an informed consent form.

The data collection tool used for this study was a modified and expanded version of a job satisfaction survey developed by Bellingham [15]. The original version includes 30 positively worded statements, for each of which respondents are required to tick a box under the “yes” or “no” columns. For the purposes of this study, interns were asked to indicate their level of agreement with each statement using a five-point Likert scale, with 1 indicating “strongly disagree” and 5 indicating “strongly agree”. Each questionnaire prompt was worded so that a higher magnitude of agreement with the statement corresponded to a higher Likert score and a higher level of satisfaction with the specific issue addressed in the statement. Mean satisfaction scores were calculated for each prompt in order to quantitatively compare the different aspects of job satisfaction. These mean scores also provided data used to quantify overall job satisfaction among the participants.

Some of the statements from the 30-item Bellingham survey were modified or substituted, either so that they more specifically referred to doctors, or in order for there to be an equal number of prompts (three) in each of the ten categories to be considered as individual satisfaction-influencing factors in the data analysis. The categories and replacement statements were extracted from the literature review [1, 2, 5, 6, 16]. The categories used in this study were (1) *hours and pay*, (2) *work content*, (3) *working conditions*, (4) *relationships with senior staff members*, (5) *workplace relationships with peers*, (6) *educational environment*, (7) *internal factors* (those primarily influenced by the respondent's own attitudes and values), (8) *quality of life*, (9) *autonomy and perception of self-worth at work*, and (10) *future career prospects*. Mean Likert scale response scores ("mean satisfaction scores") were calculated for each category and Student's t-test was used to compare each category-specific mean satisfaction score to the others. Student's t-test (or ANOVA, where applicable) was also used to compare the mean satisfaction scores corresponding to each respective stratum amongst the sociodemographic variables that were investigated (sex, age, marital status, medical school qualification route, medical school completion year, and hospital affiliation).

The 30 items from the Bellingham survey accounted for one section (section C) of the six sections of the full questionnaire used for this study. Section (A) asked for sociodemographic data; section (B) consisted of two yes/no questions explicitly asking the interns to indicate if they were committed to and satisfied with their jobs; section (D) repeated the questions from section (B) in case respondents may have changed their minds while considering their responses in the previous section. In section (E), participants were asked to list the top five statements, from the 30 items in section (C), in descending order of importance in terms of their influence on their own level of job satisfaction and job commitment. The responses gathered in section (E) allowed for ordered ranking of the individual statements and categories (from section C) in terms of their overall influence on the interns' job satisfaction and commitment. Frequencies were calculated for each section (C) statement and, by extension, for each category, by indiscriminately tallying the number of times each statement was listed as first, second, third, fourth, or fifth most important. Weighted frequency scores were calculated for each statement and category by assigning a weighting factor, arbitrarily ranging from one to five, to each listed statement before tallying, depending on whether it was identified as the fifth, fourth, third, second, or most influential statement, respectively. Section (F) was similar to section (E) in that it prompted respondents to list the top five most influential items in section (C). Instead of ranking questionnaire prompts according to their influence on job satisfaction and commitment, however, section (F) asked participants to list the five most important section (C) items in descending order of their influence on their agreement or disagreement with the decision to strike in late 2010 and early 2011.

Questionnaire responses were compiled using a Microsoft Excel 2007 database and quantitative analysis was done using Epi Info 3.5.3.

## Results

### Sociodemographic data

Of the 70 interns who were identified as working at QECH or KCH and given questionnaires, 61 (87.1%) returned their questionnaires either fully completed or nearly fully completed. Sociodemographic data of the respondents are shown in Table 1. The majority of respondents were male (65.6%), aged 21 to 24 years (61.0%), single (93.4%), and used the COM premedical sciences programme to qualify for medical school (83.6%). There was a nearly equal split of participants from each COM graduation cohort (32 graduated in 2010 and 29 in 2011) and from each hospital (31 from QECH, 30 from KCH) included in the study. The mean age of the study participants was 24.4 years; the youngest intern was 21 years of age, the eldest was 31 years, and two of the respondents did not disclose their ages. Notably, there were no significant differences in mean satisfaction score amongst the various strata in any of the investigated sociodemographic variables (Table 1).

**Table 1:** sociodemographic data of medical interns at QECH and KCH in December

Characteristic		Number (n = 61)	Percent (%)	Mean satisfaction score	sd	p-value
**Sex**	Male	40	65.6	3.45	0.47	0.30
	Female	21	34.4	3.31	0.59	
**Age (years; mean 24.4)**	21-24	36	59	3.41	0.53	0.59
	25-31	23	37.7	3.42	0.5	
	Not reported	2	3.3	3.03	0.24	
**Marital status**	Single	57	93.4	3.38	0.51	0.41
	Engaged	2	3.3	3.75	0.26	
	Married	2	3.3	3.72	0.74	
**Medical school entry qualification**	COM premed	51	83.6	3.39	0.51	0.15
	A-level	8	13.1	3.62	0.46	
	BSc	2	3.3	2.85	0.5	
**Medical school completion year**	2010	32	52.5	3.39	0.54	0.86
	2011	29	47.5	3.42	0.49	
**Hospital affiliation**	QECH	31	50.8	3.39	0.54	0.80
	KCH	30	49.2	3.42	0.49	
**Total**		**61**		**3.40**	**0.51**	

QECH = Queen Elizabeth Central Hosptial, Blantyre; KCH =Kamuzu Central Hospital, Lilongwe; COM premed = College of Medicine premedical sciences programme; sd = standard deviation; Student’s t-test and ANOVA were used to generate p-values

### Overall job satisfaction and commitment

The interns were asked explicitly whether or not they were satisfied with, and committed to, their jobs twice in the questionnaire: before and after the 30 statements in section (C). Forty-two (68.9%) of the 61 interns initially indicated that they were satisfied. The proportion of satisfied interns fell to 34 (56.7%) of 60 respondents after section (C).

The majority of interns indicated that they were committed to their jobs, with 97.0% of the respondents identifying themselves as committed both before and after section (C) (59 of 61 respondents then 58 of 60 respondents, respectively).

The overall mean satisfaction score for all participants was 3.40 ± 0.51. The mean satisfaction score for those who identified themselves as satisfied after the first instance of asking was 3.55 ± 0.47, and the mean satisfaction score for the interns who declared themselves dissatisfied was 3.07 ± 0.45, with the difference being significant at the 99% confidence level (p = 0.0004). These statistics were nearly identical when the “yes” group from the second instance of asking was compared to the “no” group (mean_yes_= 3.64 ± 0.43, mean_no_= 3.09 ± 0.45, p << 0.01).

### Factors influencing intern job satisfaction

**Figure 1:**
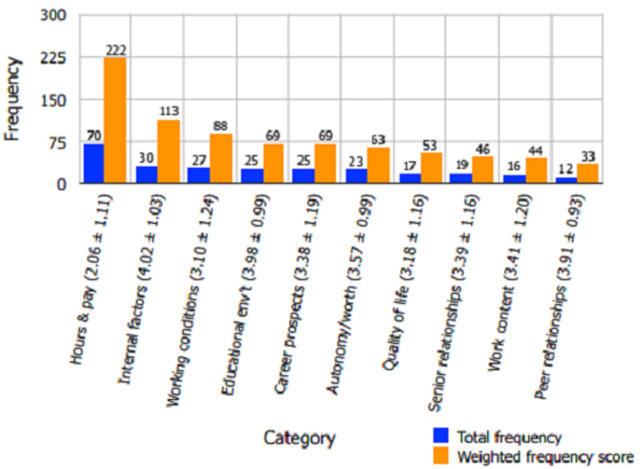
factors ordered in terms of their influence on Malawian medical intern job satisfaction

**Figure 2:**
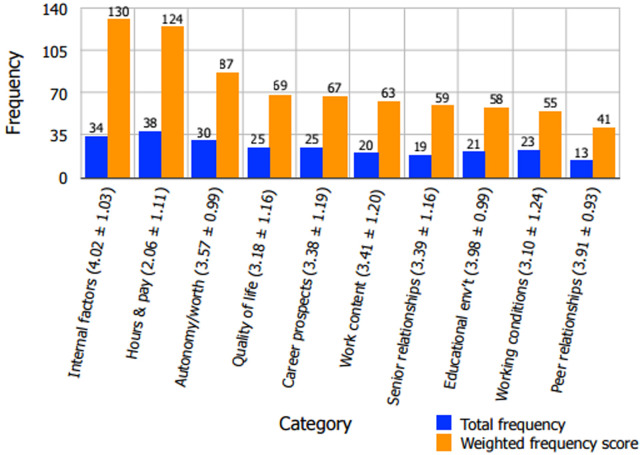
factors ordered in terms of their influence on Malawian medical intern job commitment

**Figure 3:**
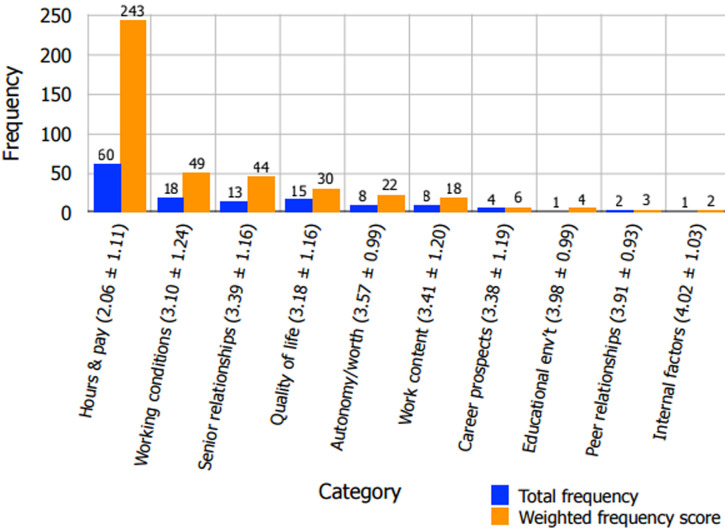
factors ordered in terms of their influence on Malawian intern justification of the 2010-2011 strike

Of the categories investigated, *hours and pay* (total frequency 70, weighted frequency score 222) was most frequently cited as strongly influencing job satisfaction. *Hours and pay*also yielded the lowest mean satisfaction score (2.06 ± 1.11). Figure 1 shows the categories, arranged in descending order from left to right, in terms of their corresponding satisfaction-related weighted frequency scores. Figure 1 also displays the mean satisfaction scores for each category (as do Figure 2 and Figure 3). *Workplace relationships with peers* (total frequency 12, weighted frequency score 33) was the least influential job satisfaction-determining factor. *Internal factors* (mean satisfaction score 4.02 ± 1.03), *educational environment* (mean satisfaction score 3.98 ± 0.99), and *workplace relationships with peers* (mean satisfaction score 3.91 ± 0.93) were the three categories with the highest mean satisfaction scores. Table 2 shows that “I earn an appropriate wage for the amount of time that I work” (total frequency 38, weighted frequency score 135, mean satisfaction score 1.72 ± 1.02) was the single questionnaire prompt most frequently listed as strongly influencing job satisfaction; it was also the statement with the lowest mean satisfaction score. Of the three questionnaire statements most frequently cited as markedly affecting job satisfaction, two were from the *hours and pay*category (Table 2).

**Table 2:** questionnaire statements listed in descending order of their influence on Malawian intern job satisfaction

Statement	Category	Total frequency	Weighted frequency score	Mean satisfaction score (sd)
I earn an appropriate wage for the amount of time that I work.	Hours and pay	38	135	1.72 (1.02)
I feel proud to be a doctor.	Internal factors	20	78	4.62 (0.73)
My working hours are reasonable.	Hours and pay	20	59	2.42 (1.25)
I have the materials and equipment that I need in order to do my work right.	Working conditions	17	57	2.20 (1.09)
I have opportunities to learn what I want to learn.	Educational environment	16	48	3.72 (1.07)
I am satisfied with my future career prospects.	Future career prospects	14	37	3.85 (1.08)
I feel positive and up most of the time I am working.	Internal factors	8	32	3.67 (1.03)
I feel recognized and appreciated at work.	Autonomy and self-worth	10	31	3.70 (0.95)
I am fairly compensated.	Hours and pay	12	28	2.05 (0.95)
I’m engaged in meaningful work.	Work content	9	28	4.26 (0.63)
Work is a real plus in my life.	Quality of life outside of work	7	25	3.74 (0.87)
I have good friends at work.	Workplace relationships with peers	9	24	4.21 (0.78)
I feel free to be who I am at work.	Autonomy and self-worth	8	22	3.43 (1.19)
I trust our leadership team.	Relationships with senior staff	8	19	3.65 (1.10)
I have energy at the end of each workday to engage in personal interests.	Quality of life outside of work	6	17	2.87 (1.17)
My manager cares about me as a person.	Relationships with senior staff	5	17	3.08 (1.16)
I regularly encounter situations that reinforce my commitment to being a doctor for a long time to come.	Future career prospects	6	16	3.51 (1.13)
I know what is expected of me at work.	Working conditions	5	16	3.79 (0.99)
I have been able to obtain useful career advice since graduation.	Future career prospects	5	16	2.77 (1.10)
I feel informed about what’s going on at work.	Working conditions	5	15	3.30 (1.07)
I am rarely expected to perform routine work that can be done by staff without medical qualifications.	Work content	6	13	2.83 (1.26)
I learn something new every day at work.	Educational environment	5	12	4.20 (0.91)
I have energy at the end of each workday to attend to the people I care about.	Quality of life outside of work	4	11	2.92 (1.20)
My manager reviews my progress.	Relationships with senior staff	6	10	3.45 (1.17)
My opinions count.	Autonomy and self-worth	5	10	3.59 (0.78)
I find Journal Club meetings, Grand Rounds, etc. useful.	Educational environment	4	9	4.02 (0.94)
My coworkers are committed to doing quality work.	Workplace relationships with peers	3	9	3.26 (0.95)
I look forward to going to work on Monday morning.	Internal factors	2	3	3.75 (1.03)
I am rarely expected to perform clinical tasks for which I do not feel adequately trained.	Work content	1	3	3.12 (1.12)
I respect the work of my peers.	Workplace relationships with peers	0	0	4.25 (0.70)

“Total frequency” represents the total number of times each questionnaire statement was listed by respondents as one of the five most important satisfaction-influencing questionnaire statements. “Weighted frequency score” represents tally if the number of times an item was listed as most important is multiplied by 5, number of times listed as second most important is multiplied by 4, number of times listed as third most important is multiplied by 3, etc. Higher frequency and weighted frequency scores are analogous to greater importance. Mean satisfaction score possibilities range from 1.0 to 5.0, with higher scores corresponding to higher magnitude of agreement with the respective questionnaire statements.

sd = standard deviation

### Factors influencing intern job commitment

*Internal factors* (total frequency 34, weighted frequency score 130) and *hours and pay* (total frequency 38, weighted frequency score 124) were found to be the most influential factors on the interns' levels of job commitment (Figure 2). “I feel proud to be a doctor” (total frequency 22, weighted frequency score 82), from the *internal factors*category, was the questionnaire item most often identified as greatly impacting intern job commitment (Table 3); it also yielded a mean satisfaction score of 4.62 ± 0.73, which was the highest among all of the statements (Table 2). The *workplace relationships with peers*category (total frequency 13, weighted frequency score 41) was found to have the least impact on intern job commitment (Figure 2).

**Table 3:** five most important questionnaire items in terms of their influence on intern job commitment

Statement	Category	Total frequency	Weighted frequency score	Mean satisfaction score (sd)
I feel proud to be a doctor.	Internal factors	22	82	4.62 (0.73)
I earn an appropriate wage for the amount of time that I work.	Hours and pay	17	55	1.72 (1.02)
I feel recognized and appreciated at work.	Autonomy/worth	14	45	3.70 (0.95)
I'm engaged in meaningful work.	Work content	12	44	4.26 (0.63)
My working hours are reasonable.	Hours and pay	12	42	2.42 (1.25)

“Total frequency” represents the total number of times questionnaire items were listed by respondents in personal top-five most influential factors. “Weighted frequency score” represents tally if the number of times an item was listed as most important is multiplied by 5, number of times listed as second most important is multiplied by 4, number of times listed as third most important is multiplied by 3, etc. Higher frequency and weighted frequency scores are analogous to greater importance. Mean satisfaction score possibilities range from 1.0 to 5.0, with higher scores corresponding to higher magnitude of agreement with the respective questionnaire statements.

sd = standard deviation

### Factors influencing the 2010-11 QECH intern strike

Of the 47 interns who responded to the question, “Did you agree with the decision to strike?” in section (F) of the questionnaire, 39 (83.0%) answered in the affirmative. In terms of influence on these respondents' justification of the 2010-2011 intern strike at QECH, *hours and pay* (total frequency 60, weighted frequency score 243) was found to be more important than all other categories (Figure 3, Table 4). Each category examined in this study was represented by three statements in section (C) of the questionnaire, and the three statements in the *hours and pay*category (“I earn an appropriate wage for the amount of time that I work”, “I am fairly compensated”, and “My working hours are reasonable”) were the three most commonly cited statements when interns were asked to list the top five strike-justifying questionnaire items. At the bottom of the list, few respondents indicated that *career prospects*(total frequency 4, weighted frequency score 6, mean satisfaction score 3.38 ± 1.19), *educational environment*(total frequency 1, weighted frequency score 4), *workplace relationships with peers*(total frequency 2, weighted frequency score 3), and *internal factors*(total frequency 1, weighted frequency score 2) were important factors in developing an opinion about the rationale of the strike (Figure 3).

**Table 4:** five most important questionnaire items in terms of influence on the 2010-11 Malawian intern strike

Statement	Category	Total frequency	Weighted frequency score	Mean satisfaction score (sd)
I earn an appropriate wage for the amount of time that I work.	Hours and pay	27	123	1.72 (1.02)
I am fairly compensated.	Hours and pay	17	66	2.05 (0.95)
My working hours are reasonable.	Hours and pay	16	54	2.42 (1.25)
I have the materials and equipment that I need in order to do my work right.	Working conditions	13	39	2.20 (1.09)
I trust our leadership team.	Relationships with senior staff	7	22	3.65 (1.10)

“Total frequency” represents the total number of times questionnaire items were listed by respondents in personal top-five most influential factors. “Weighted frequency score” represents tally if the number of times an item was listed as most important is multiplied by 5, number of times listed as second most important is multiplied by 4, number of times listed as third most important is multiplied by 3, etc. Higher frequency and weighted frequency scores are analogous to greater importance. Mean satisfaction score possibilities range from 1.0 to 5.0, with higher scores corresponding to higher magnitude of agreement with the respective questionnaire statements.

sd = standard deviation

## Discussion

To alleviate what was considered a health-sector human resources crisis in 2005, the Malawi government initiated a six-year Emergency Human Resources Programme (EHRP), which, among other interventions, included salary increases that helped make health workers the highest paid civil servants in the country at the time [16, 17]. Despite the salary increases, junior doctors in Malawi still considered remuneration the least satisfying (and most important) aspect of their job soon after the six years of the EHRP elapsed. The dissatisfaction remained even though the EHRP was summarised as a success in the final report [18]. If the EHRP was indeed effective, the results of this study suggest that the efforts need to not only continue, but also intensify.

Salary, working hours, and physical working conditions were also found to be the lowest rated aspects of the job in a 2005 survey of psychological morbidity among medical interns in Ilorin, Nigeria [1]. As with our study, the investigators in Nigeria also found that “intrinsic” factors (what we termed *internal factors*) were among the job satisfaction determinants that interns were most pleased with. Knowledge of the satisfaction levels corresponding to each of the individual job satisfaction determinants becomes more useful with the addition of an awareness of the prevailing perceived importance of each of these determinants amongst the interns. We have therefore gone one step further than the Nigerian study by establishing the importance of these factors among Malawian medical interns in addition to their mean Likert scale ratings.

The wording of the 30 questionnaire statements in section (C) makes it reasonable to translate the Likert scale used for this study to mean that a response of 1 corresponded to “extremely dissatisfied” and a response of 5 corresponded to “extremely satisfied”. Keeping this in mind, it is noteworthy that the *hours and pay*category generated, by a substantial margin, the lowest mean satisfaction scores (analogous to satisfaction levels) and the highest frequency scores (analogous to importance). It is also noteworthy that the four statements with the highest frequency scores in terms of their influence on satisfaction (Table 2) produced the relatively low cumulative mean satisfaction score of 2.74. It follows then, that the overall cumulative mean satisfaction score of 3.40 likely overestimates the Malawian interns' overall job satisfaction level. In other words, the unweighted mean satisfaction scores, as reported above, only achieve their true validity as measures of satisfaction in the context of their respective frequency scores and weighted frequency scores. Without weighting, the mean satisfaction scores imply the assumption that all of the questionnaire items were of equal importance to all of the interns, which was predictably and evidently untrue. The mean satisfaction scores were further validated by the significant difference between the scores of interns who identified themselves as satisfied and those who identified themselves as dissatisfied when asked directly.

### Study limitations

The analysis of results in this study was entirely quantitative. No open-ended questions were posed to the study participants, and this may be considered a limitation of the study. Some qualitative data would have undoubtedly enriched our results and analysis, but our conclusions can nonetheless be used to design future qualitative research that focuses more keenly on the factors highlighted by this study as important. Quantitative analysis was favoured to facilitate more straightforward and structured presentation of the results. The quantitative approach also allowed for a relatively short questionnaire, which likely contributed to the high participation rate. Another limitation of this study was that it relied solely on the literature to determine the factors that were addressed in the questionnaire. It was clear, while listening to some of the comments made by the interns who completed their questionnaires in our presence, that important satisfaction-influencing and strike-influencing factors (most notably issues dealing with intern housing) were overlooked. We recommend future qualitative research, perhaps using open-ended questionnaires, in-depth interviews, or focus group discussions, specifically dealing with the most important intern job satisfaction determinants, as revealed by this study: working hours, remuneration, and physical working conditions.

## Conclusion

We suggest that future research and interventions aimed at improving intern job satisfaction in Malawi focus on assessing and improving the remuneration, working hours, and physical working conditions of medical interns.
